# Reviews

**Published:** 1882-10

**Authors:** 


### Reviews.

**Essentials of Vaccination.** A Compilation of Facts Relating to Vaccine Inoculation and its Influence in the Prevention of Small-pox. By W. A. HARDWAY, M. D., Professor of Diseases of the Skin in the Post-Graduate Faculty of the Mo. Med. College, etc., St. Louis. Chicago: Jansen, McClurg & Co. 1882.

Dr. Hardway has succeeded in producing a very interesting book, containing a great deal of useful information. It is not intended as a comprehensive treatise on vaccination, but the author modestly claims it to be "a careful compilation of the most essential facts relating to the subject." He treats of the History of Vaccination; Variola in Animals; Nature of Vaccinia; Vaccinia in the Human Subject; Abnormal Modifications and Complications; Re-vaccination; Merits of Different Kinds of Virus; Methods of Obtaining and Storing; the Operation of Vaccinating, and a Brief Review of the Objections to Vaccination. The author's claim for it is abundantly fulfilled, and the book will, no doubt, prove acceptable to the profession.

---

**Labor Among Primitive Peoples.** Showing the Development of the Obstetric Science of To-day, from the Natural and Instinctive Customs of all Races, Civilized and Savage, Past and Present. By GEO. J. ENGLEMAN, A. M., M. D., Professor of Obstetrics in the Post-Graduate School of the Missouri Medical College, etc. St. Louis: J. H. Chambers & Co. Cloth 8vo, pp. 202. Price, $2.

The amount of labor and research involved in the production of this work must have been very great. Some of the chapters have appeared before in the *American Journal of Obstetrics*, and other journals, but the subject is so interesting to the physician, and, in a certain sense, important, that their preservation in book form is a subject for congratulation. It is interesting to observe how similar the obstetric practices of primitive people are to our own, and in those cases in which the hands and external manipulation alone were sufficient, there is evidence of expertness not inferior to that of to-day; indeed, we will find much to study, to imitate and to develop. The book is well illustrated and is in every way a credit both to the author and publisher.

**A Treatise on the Science and Practice of Medicine; or, the Pathology and Therapeutics of Internal Diseases.** By ALONZO B. PALMER, M. D., LL. D., Professor of Pathology and Practice of Medicine and of Clinical Medicine in the University of Michigan, etc., etc. Volume II. New York: G. P. Putnam's Son, 27 and 29 West Twenty-third Street. 1882.

The present volume treats of diseases of the respiratory system, diseases of the heart, diseases of the urinary apparatus and urinary diseases, diseases of the male sexual system, diseases of the brain and nervous system, diseases of the mind, and parasitic diseases. It is from the pen of one of the most industrious writers in the profession, and in its arrangement shows commendable care, labor and ability, not only in its details but in the general treatment of the subjects. We recognize, in reading its pages, a familiar style to which, many years ago, we were accustomed to listen, and the subject-matter, especially the therapeutics, presents the author's views as they were given to the medical class of which we were once a member. The work, therefore, possesses more than ordinary interest to the writer, and in its perusal and examination we have been turned from the spirit of severe criticism, not only by the superior merits of the volume, but by the appreciation always entertained for the ability and wide experience of the author. We regard the work as a valuable addition to our medical literature. The author has enjoyed a rare experience in diseases peculiar to the West, and the profession, in that section especially, will find the work a most important guide in the pathology and treatment of diseases the type of which is modified by climatic influences. We cannot enter upon a critical review of the author's opinions of morbid phenomena, nor of the manner in which he presents them. Prof. Palmer's reputation is a sufficient guarantee of the value of the production he has given to the profession. We warmly endorse the work, and are assured that it will fill an important position in the libraries of medical men.—L.

